# Clinically-Relevant ABC Transporter for Anti-Cancer Drug Resistance

**DOI:** 10.3389/fphar.2021.648407

**Published:** 2021-04-19

**Authors:** Huan Xiao, Yongcheng Zheng, Lingling Ma, Lili Tian, Qiu Sun

**Affiliations:** ^1^ State Key Laboratory of Biotherapy, Cancer Center, West China Hospital, Sichuan University and Collaborative Innovation Center for Biotherapy, Chengdu, China; ^2^ Department of Anesthesiology, Sichuan Provincial People’s Hospital, University of Electronic Science and Technology of China, Chengdu, China

**Keywords:** ABCC1, ABCG2, ABCB1, MDR, anti-cancer

## Abstract

Multiple drug resistance (MDR), referring to the resistance of cancer cells to a broad spectrum of structurally and mechanistically unrelated drugs across membranes, severely impairs the response to chemotherapy and leads to chemotherapy failure. Overexpression of ATP binding cassette (ABC) transporters is a major contributing factor resulting in MDR, which can recognize and mediate the efflux of diverse drugs from cancer cells, thereby decreasing intracellular drug concentration. Therefore, modulators of ABC transporter could be used in combination with standard chemotherapeutic anticancer drugs to augment the therapeutic efficacy. This review summarizes the recent advances of important cancer-related ABC transporters, focusing on their physiological functions, structures, and the development of new compounds as ABC transporter inhibitors.

## Introduction

Multidrug resistance (MDR) refers to the resistance of a wide spectrum of structurally and mechanistically unrelated drugs across the membrane. This process is among the culprits of failure of cancer chemotherapy, since the cancer cells can efflux chemotherapy agents and therefore reduce the intracellular drug levels (Ahmed et al., 2020). Members of the ATP-binding cassette family have been found to be involved in this process. To be specific, the ABC transporter family can be divided into seven subfamilies according to their genome sequences and TMDs (transmembrane domain) structures (Taylor et al., 2017). Some of them have been reported to act both as importers and exporters of bacteria, however, in eukaryotic cells, they all efflux pumps (Robey et al., 2018). P-glycoprotein (P-gp) was the first identified member within this family and a high-resolution structure of the mouse homolog, which has 87% sequence identity with human has been elucidated (Juliano and Ling, 1976). Except for P-gp, ABCC1(also known as MRP1)and ABCG2 (also known as BCRP) have also been extensively studied (Toyoda et al., 2019; Ambjørner et al., 2020), confirming their prominent roles in multidrug resistance of cancer cells. During the past few decades, numerous efforts have been made to solve the drug resistance caused by these transporter proteins. However, most of these attempts lead to disappointing results for both the first generation and the second generation of inhibitors, because they exhibit either unacceptable levels of toxicity or less potential inhibiting effects (Adamska and Falasca, 2018). So far, more inhibitors have been exploited (from nature or synthetic sources Gonçalves et al., 2020). In addition, researchers have achieved a deeper understanding of the phenomenon of chemotherapy resistance through their concerns to the genes and signaling pathways that modulate the expression of these proteins (Li et al., 2018; Sultan et al., 2018; Huang et al., 2020; Zhou et al., 2020). In this review, we summarize the recent progress of the most clinically significant ABC transporters ABCB1, ABCG2, and ABCC1 that cause multi-drug resistance during cancer therapy, with the emphasis on novel small molecule compounds that are tested in preclinical and clinical studies, mainly on natural products, synthetic compounds, aiming to provide a wider perspective to understand the multidrug resistance and new strategies targeting ABC transporters in cancer treatment.

## Locations, Substrates, Cancer Type

### ABCB1 (P-gp)

P-gp was the first found human ABC transporter of all known ones and was identified as a glycoprotein responsible for modulating drug permeability (Juliano and Ling, 1976). MDR, the gene encoding P-gp, is located in chromosome 7 at q21 and contains 28 exons encoding 1280 amino acids of this 170 kDa protein (Gottesman et al., 1995). P-gp are similarly expressed in human and mouse tissues, with a more biased expression in excretory tissues, including breast, blood-brain barrier, liver, pancreas, and kidney, and in the apical membrane of epithelial cells located at physiological barriers (Sita et al., 2017; Wang et al., 2017; Christie et al., 2019; Zhang et al., 2020). P-gp exports neutral or positively charged hydrophobic compounds and xenobiotics from cells, thereby protecting them from cytotoxicity (Sharom, 2011; Fletcher et al., 2016). The critical role of P-gp in the blood-brain barrier (also known as BBB), was first illustrated by Schinkel et al. (Schinkel et al., 1994). They found the deletion of *Abcb1a* and *Abcb1b* can lead to CNS toxicity from ivermectin, however, despite its defensive role in protecting cells, the overexpression of P-gp mRNA and protein in clinical specimens in breast, kidney, and lung cancers portends a poor response to chemotherapy, resulting in low survival rates (Robey et al., 2010; Amiri-Kordestani et al., 2012). P-gp can efflux chemotherapy agents and reduce intracellular drug levels (Ahmed et al., 2020), which is one of the major causes of chemo-resistance. The major substrates involved in the multidrug resistance of P-gp are structurally and mechanistically unrelated drugs (Abdallah et al., 2015; Yu et al., 2016; Bugde et al., 2017; Gameiro et al., 2017; Lu et al., 2017). Moreover, P-gp is preferable to express in poorly differentiated and most invasive cells (Ohtsuki et al., 2007; Mesraoua et al., 2019). In a range of soft tissue sarcomas, P-gp expresses most in the largest and most aggressive tumors (Oda et al., 2005). Single-nucleotide polymorphisms (SNP) occurring in *ABCB1* genes can result in increased or decreased transporter efficacy, depending on the gene type of the variants, which remains complex so far (Dulucq et al., 2008; Zu et al., 2014).

### ABCG2

ABCG2 plays a pivotal role in extruding exogenous and endogenous substrates and drugs (Ando et al., 2007; Chen YL et al., 2016; Halwachs et al., 2016; Gewin et al., 2019; Mares et al., 2019; Orlando et al., 2019; Traxl et al., 2019), which is related to many multidrug resistant cancer cell lines, including acute lymphoblastic leukemia (ALL), retinal progenitors, hepatic metastases, gastric carcinoma, fibrosarcoma, nonsmall cell lung cancer, glioblastoma and myeloma (Natarajan et al., 2012; Olarte Carrillo et al., 2017; Abdel Gaber et al., 2018; Reustle et al., 2018; Zhang et al., 2018). ABCG2 locates in the plasma membrane of the cell and expresses in normal tissues like placenta, prostate, kidney, blood-brain barrier, liver, ovary, small intestine, and seminal vesicle (Jackson et al., 2018), which is responsible for regulating the intracellular levels of hormones, lipids, ion and intracellular organelles such as mitochondrion (Ding et al., 2019), lysosome (Chapuy et al., 2008), endoplasmic reticulum (Kashiwayama et al., 2009), Golgi apparatus (Tsuchida et al., 2008). ABCG2 also has a wide range of mechanistically and structurally different substrates, such as mitoxantrone, methotrexate, camptothecins, topotecan and irinotecan, SN-38, epipodophyllotoxin, imidazoacridinones, the anthracycline doxorubicin (Bram et al., 2009a; Bram et al., 2009b; Mao and Unadkat, 2015) and tyrosine kinase inhibitors (Dohse et al., 2010; Hegedüs et al., 2012). ABCG2 has a less important role in uric acid transport, however, its dysfunction leads to several diseases linked to hyperuricaemia such as gout, kidney disease, and hypertension (Bram et al., 2009b; Ishikawa et al., 2013). What is more, phytoestrogen sulfate conjugates (Wetering and Sapthu, 2012), uremic toxin, and indoxyl sulfate (Takada et al., 2018) are unique substrates of ABCG2. A genetically engineered mouse model about BRCA1-associated breast cancer (Brca1−/−p53−/− mice) has identified that ABCG2 overexpression is the cause of acquired topotecan resistance, and the genetic ablation of ABCG2 improves the survival rate of topotecan-treated animals (Zander et al., 2010). In fact, in some cancer cell lines, more than one ABC transporter is overexpressed. High levels of ABCG2, ABCB1, and ABCC1 have been found within primitive leukemic CD34+/38- cells (Raaijmakers et al., 2005). The co-expression contributes to multidrug resistance, which requires multi-transporter inhibitors to achieve a better clinical outcome (Robey et al., 2010). However, although the ABCG2-involved multidrug resistance mechanisms are basically clear, the clinical trial relevant to ABCG2 inhibitors has received few satisfying results (Fletcher et al., 2016).

### ABCC1

ABCC1 was identified in 1992 from human small-cell lung cancer cell lines whose drug resistant behavior occurred without the overexpression of P-gp (Cole et al., 1992). ABCC1 expresses in the plasma membrane of some normal tissues and cells including liver, kidney, lung, intestine, blood-brain barrier and peripheral blood monocellular cells (Uhlén et al., 2015). Overexpression of ABCC1 is related to endometria, acute myeloblastic, glioma, lymphoblastic leukemia, head and neck, non-small cell lung cancer, neuroblastoma, melanoma, prostate, breast, renal, thyroid cancer (Cole, 2014; Johnson and Chen, 2017; Emmanouilidi et al., 2020; Si et al., 2020). To be specific, ABCC1 is a lipophilic anion pump, conferring resistance to anti-cancer drugs (Cole, 2014). Compared with P-gp, the substrates of ABCC1 have more diverse structures and most of them are amphipathic organic acids with large hydrophobic groups (Kumar and Jaitak, 2019). Endogenous substrates are mainly pro-inflammatory molecules such as Leukotrienes C4 (LTC4), hormones such as estrogens and prostaglandins, sphingosine-1-phosphate, antioxidants like glutathione and glutathione disulphide (Csandl et al., 2016; Basu et al., 2017; Fallatah and Georges, 2017). Noteworthy, Glutathione (GSH) has an impact on ABCC1 transport activities (Nasr et al., 2020). ABCC1 and GSH are synergistic to some extent. They co-transport anticancer drugs such as doxorubicin, vincristine and etoposide. ABCC1 also transports GS-conjugated anions such as LTC4 and reduced GSH with low affinity and GSSG with a higher affinity (Leier et al., 1994; Drozd et al., 2016; Zhang et al., 2016; Gana et al., 2019). Exogenous substrates include many natural products like flavonoids, vincristine, daunorubicin, doxorubicin, imatinib, methotrexate and organic anions, metabolites of drugs (Zhou et al., 2008; Whitt et al., 2016). Importantly, the MYCN oncogene, a driver of tumorigenesis in neuroblastoma, can regulate ABCC1 drug transporter at the level of transcription (Weiss et al., 1997; Porro et al., 2010; Henderson et al., 2011).

## Structure and Function

The ABC transporter family is divided into 7 subfamilies according to their genome sequences and core TMDs (transmembrane domain) structures (Taylor et al., 2017). The three transporters we discuss here belong to the type III ABC system, for they all consist of 2 × 6 TMs (transmembrane helix), a striking difference between the type I ABC systems with a minimal core of 2 × 5 transmembrane helices (TMs) and type II ABC systems harboring 2 × 10 TMs (Parcej and Tampé, 2010; Braunová et al., 2019). Except from ABCC1, ABCB1 and ABCG2 are both half-transporters, working as a homodimer. Two NBDs dimerize to form two ATPase binding sites, which catalyze the ATP hydrolysis following a common mechanism: a glutamate residue interacts with hydrolytic water for the attack of the ATP phosphate (Moody et al., 2002; Oldham and Chen, 2011; Weigl et al., 2018). In NBD1 of ABCC1, the corresponding residue is not a glutamate but an aspartate whose side chain is not long enough to interact with the hydrolytic water (Geourjon et al., 2001).

### ABCB1

**FIGURE 1:**
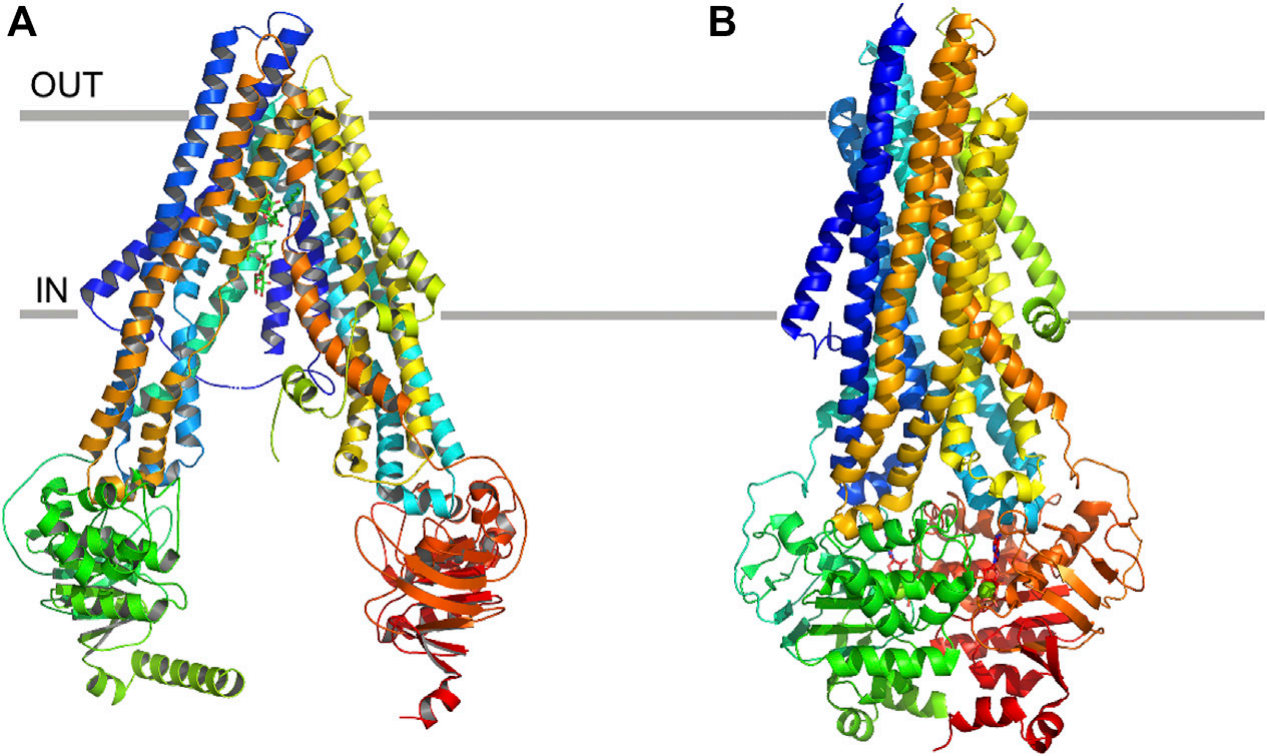
Ribbon representation of the ABCB1 structure. **(A)** apo state of ABCB1, and **(B)** ATP-binding state of ABCB1.

ABCB1 has been viewed as a “hydrophobic vacuum clearer” (Waghray and Zhang, 2018) because most of the substrates transported by this protein are hydrophobic and distributed into the lipid bilayer (Gatlik-Landwojtowicz et al., 2006). Each ABCB1 contains 1 TMD, 1 nucleotide binding domain (NBD), and forms an active transporter through dimerization. The specific binding site is located in the TMDs and the ATP hydrolysis occurs in the intracellular NBDs (Alam et al., 2019). In the apo state, the portals open to the cytoplasm and the inner leaflet of the lipid bilayer (Figure 1A). The portals are large enough to accommodate the potential substrates from the lipid bilayer and allow these hydrophobic compounds to pass through. The portals are formed by the proximity TMs (TM4/6, TM10/12). Most of the amino acid residues in the binding pocket are hydrophobic and located in the upper side of the pocket. Only 15 of the 80 residues are polar and located in the lower half of the pocket (Dawson and Locher, 2006). Different substrates or inhibitors, due to their different structures, may bind to different residues. Paclitaxel (Taxol) interacts with residues Q725, Q347, Q990 while zosuquidar interacts with M985, F982 (Alam et al., 2018; Alam et al., 2019). The conserved glutamine Q475 in NBD1, Q1118 in NBD2 can coordinate with Mg^2+^ and *gama*-phosphate of ATP, thus they play an important role in ATP hydrolysis and drug transport (Kim and Chen, 2018) (Figure 1B). In addition, tyrosine residues also play an important role as hydrogen bond donors and acceptors in ABCB1 drug transport activity. To evaluate the importance of the hydrogen bond in ligand-protein interactions, 15 conserved residues interacting with substrates are substituted with tyrosine residues. This so-called 15Y mutants can still transport small and medium size substrates, however, large substrates like Bodipy-Vinblastine cannot be normally transported. This demonstrates that in some cases it is not the hydrogen bond but the physico-chemical properties which affect the transportation (Vahedi et al., 2017).

### ABCG2

**FIGURE 2:**
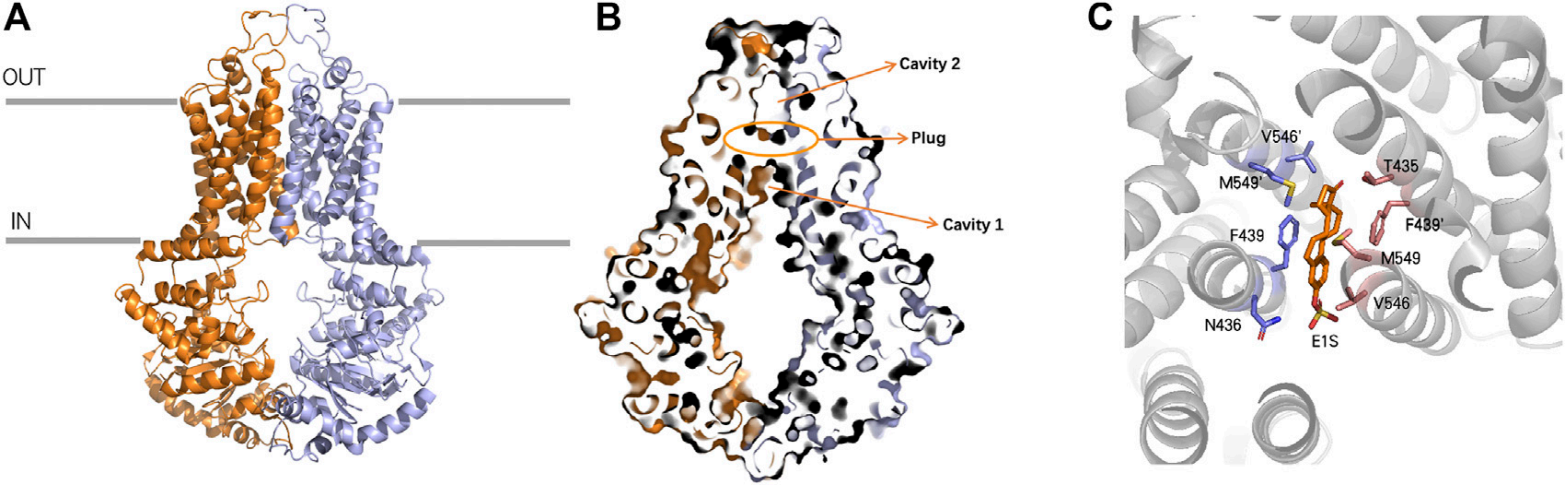
Ribbon representation of the ABCG2 structure **(A)** ribbon representation of the ABCG2 structure, with the two halves of ABCG2 colored purple and orange, respectively. **(B)** Surface representation of cavity 1, cavity 2 and Leucine plug **(C)** ABCG2 binds with E1S and the residues F439 and N436 can form stacking interactions and hydrogen bond with E1S, respectively.

ABCG2 is a half transporter, with 6 transmembrane helixes and 1 ATP-binding site. The high-resolution structure which was first elucidated in 2017 (Taylor et al., 2017; Figure 2A) brings an insight of the molecular mechanisms underlying the transport behavior. ABCG2 has 1 NBD and 1 TMD located on a single polypeptide chain and forms a homodimer as an active transporter. Unlike ABCB1 transporter, the distance between the NBDs and the membrane within the ABCG2 is smaller due to the shorter transmembrane helix and intracellular loops (Locher et al., 2002; Woo et al., 2012). The interface of TMD is formed by TM2 and TM5a from opposing ABCG2 monomers (Taylor et al., 2017). ABCG2 has two cavities involved in the transport behavior (Figure 2B). The larger cavity 1 and the smaller cavity 2 are separated by two leucine (L554, L554’) motifs (Khunweeraphong et al., 2017; Jackson et al., 2018; Manolaridis et al., 2018). Cavity 1 opens to the cytoplasm and inner leaflet of the lipid bilayer, and cavity 2 opens to the extracellular, which is located below the EL3 external loop (Khunweeraphong et al., 2019). The function of cavity 1 is to accommodate potential substrates, especially the flat, polycylic and hydrophobic ones, while cavity 2 possesses lower affinity for these substrates because of its less pronounced hydrophobic interface (Orlando and Liao, 2020). However, the lower affinity may release substrates more easily (Taylor et al., 2017). Two critical steps are involved in the process of substrate transport. Firstly, the di-leucine valve regulates the small molecules to enter the upper cavity, which is a key element for the catalytic cycle. Secondly, the essential residue E585 are harbored by the re-entry helix in the roof, making it more accessible to the extracellular (Khunweeraphong et al., 2019). The mutants N436A, F439A decrease both the substrate transport activity and ATPase activity which proves the functions of these two residues in binding and transporting estrone-3-sulfate (E1S) (Figure 2C). Moreover, the hydrogen bond between N436 and the sulfate group of E1S and the stacking interaction between the phenyl ring of F439 and the ring system of E1S are important for binding affinity. Another mutant V546F reduces the transport activity but simultaneously increases the ATPase activity, indicating that the addition of two phenyl rings at this position mimics the binding of a substrate and stimulates the ATPase activity, but can cause “clog” upon binding with E1S (Manolaridis et al., 2018).

### ABCC1

**FIGURE 3:**
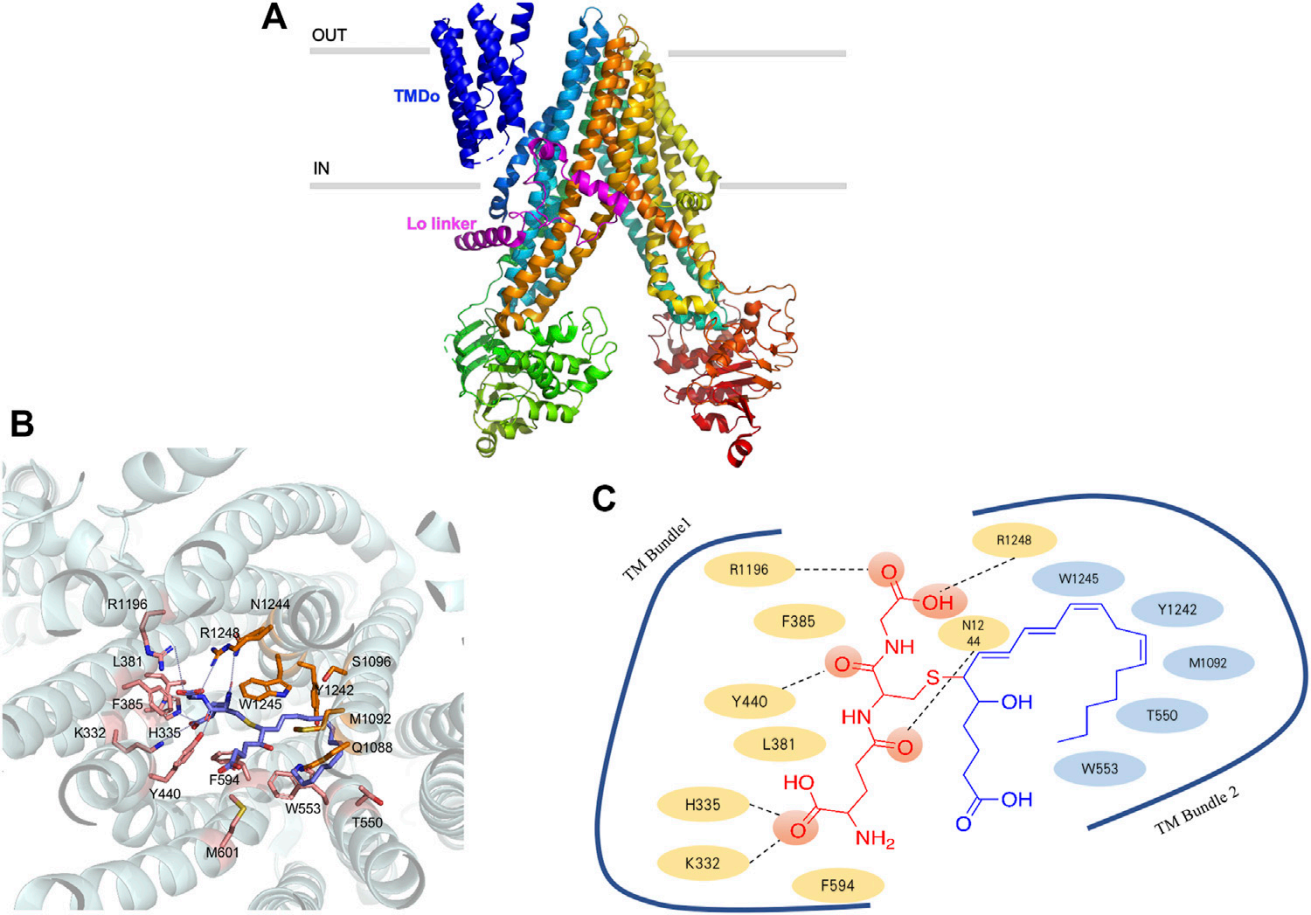
Ribbon representation of the ABCC1 structure **(A)** ribbon representation of the ABCC1 structure, with the TMDo colored dark blue and Lo linker colored purple **(B)** three-dimension of ABCC1 binding with LTC4, the proximity residues, and their interactions with LTC4 are annotated in the picture **(C)** the systematic illustration of ABCC1 binding with LTC4.

ABCC1 transporter is encoded by *ABCC1* gene, with the weight of 190 kDa and contains 1580 amino acids (Cole et al., 1992). Although there is a 23% sequence identity between P-gp and ABCC1, they have an intriguing substrate overlap. ABCC1 is a single polypeptide, containing transmembrane domains (TMDs) and two nucleotide-binding domains (NBDs) (Johnson and Chen, 2017). Only the one nucleotide-binding domain located on the NBD2 is responsible for hydrolyzing ATP and providing energy for translocation (Conseil et al., 2019). ABCC1 contains a N-terminal membrane-bound region (TMDo) domain that links to the transporter core through a Lo linker (Figure 3A). The truncation of TMDo behaves like wild-type (Bakos et al., 1998; Johnson and Chen, 2017), whereas the loss or mutation of Lo linker results in false protein folding and defective function (Bakos et al., 1998; Bakos E et al., 2000; Bakos É et al., 2000). The binding pocket of ABCC1 transporter is formed by two bundles, TM1 and TM2, and the inner-face residues provided by these two bundles are quite different (Conseil et al., 2019). Positively charged residues locate in TM1 while hydrophobic residues locate in TM2. The positive charged region usually binds with the moiety of GSH and another region rich in hydrophobic residues binds to the substrate (Johnson and Chen, 2017). LTC4 can be selectively transported by ABCC1 (Figures 3B,C) and the way in which LTC4-GSH conjugates to pass through ABCC1 has been elucidated by several studies (Loe et al., 1996; Liening et al., 2016; Conseil et al., 2019). Amphipathic substrates that contain both negatively charged and hydrophobic residues can be transported without conjugating with GSH. Besides MYCN oncogene regulates the transcription of ABCC1, the transfection of MCF-7/VP-16 breast cancer cells with miR-326 can downregulate ABCC1 expression and increase cancer cell sensitivity to etoposide and doxorubicin (Liang et al., 2010).

## ABC Transporter Inhibitors

**FIGURE 4:**
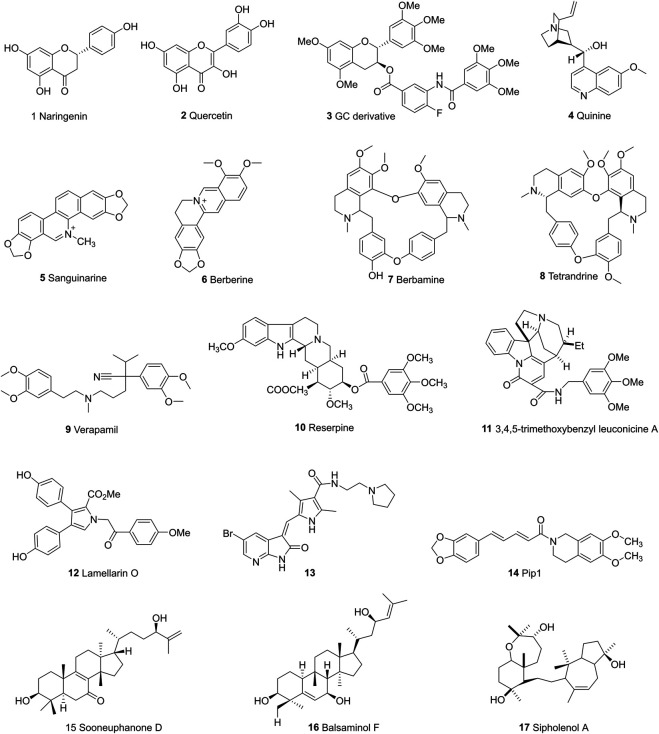
The chemical structures of representative P-gp inhibitors.

**FIGURE 5:**
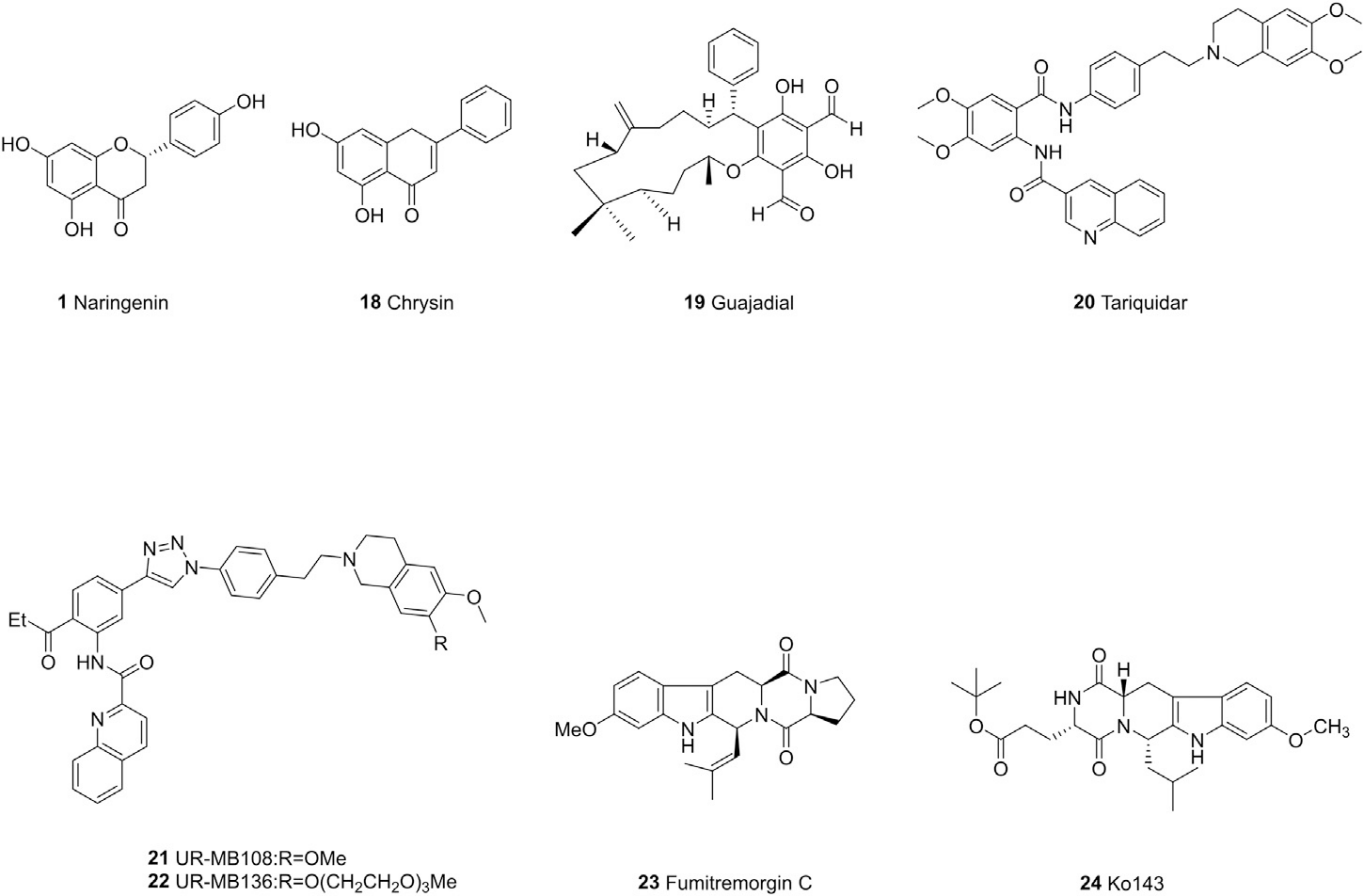
The chemical structures of representative ABCG2 inhibitors.

**FIGURE 6:**
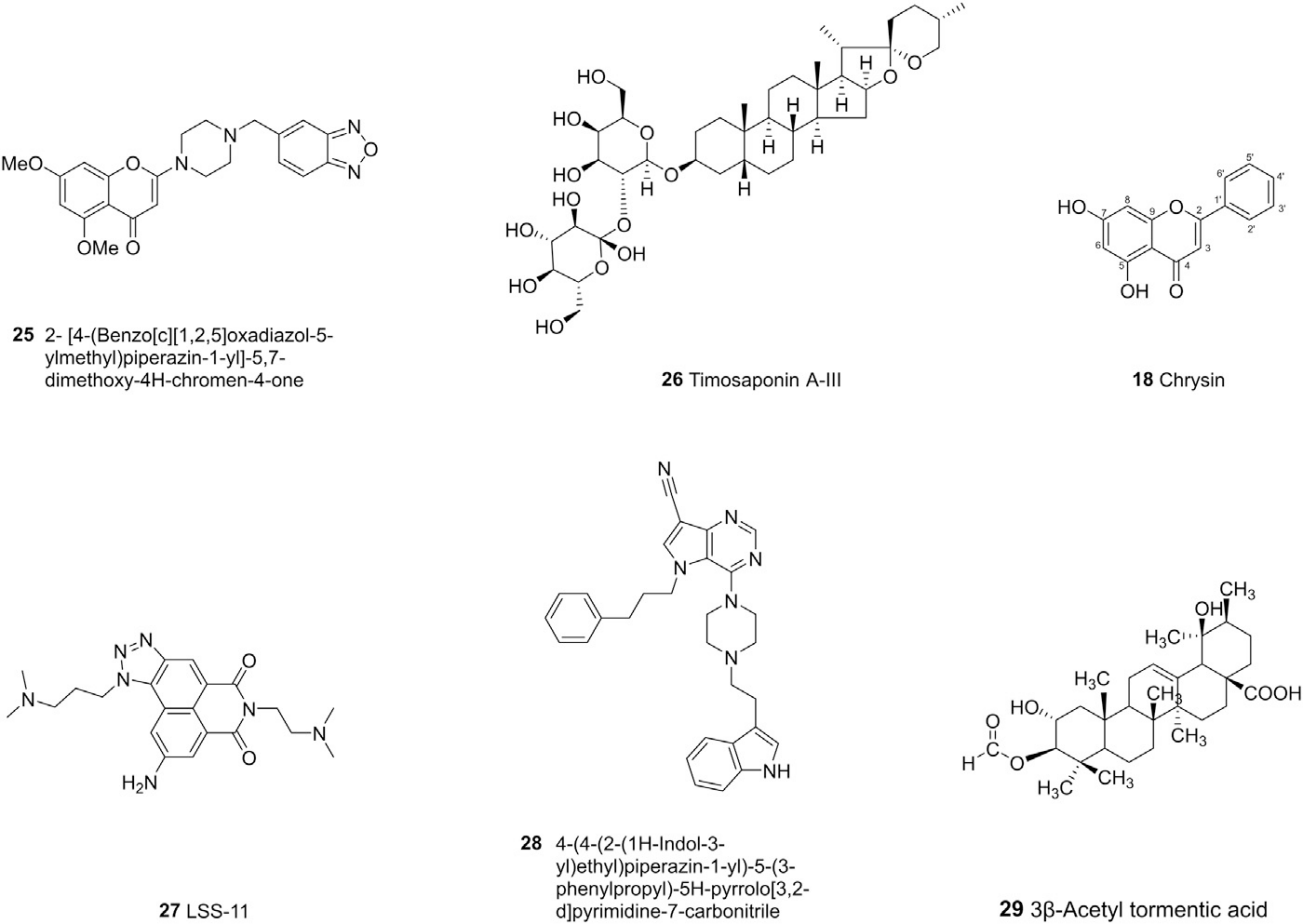
The chemical structures of representative ABCC1 inhibitors.

The past few decades have seen numerous efforts made to solve the drug resistance caused by ABC transporter proteins. Many of the first and second-generation ABC transporter inhibitors exhibit either high levels of toxicity or low potential inhibiting effects (Adamska and Falasca, 2018). Researchers are exploiting more potent inhibitors, mainly focusing on synthetic compounds and chemicals from nature plants. The chemical structures of representative ABC inhibitors are shown in Figures 4–6.

## P-Gp Inhibitors

### Natural Products

#### Flavonoids

**FIGURE 7:**
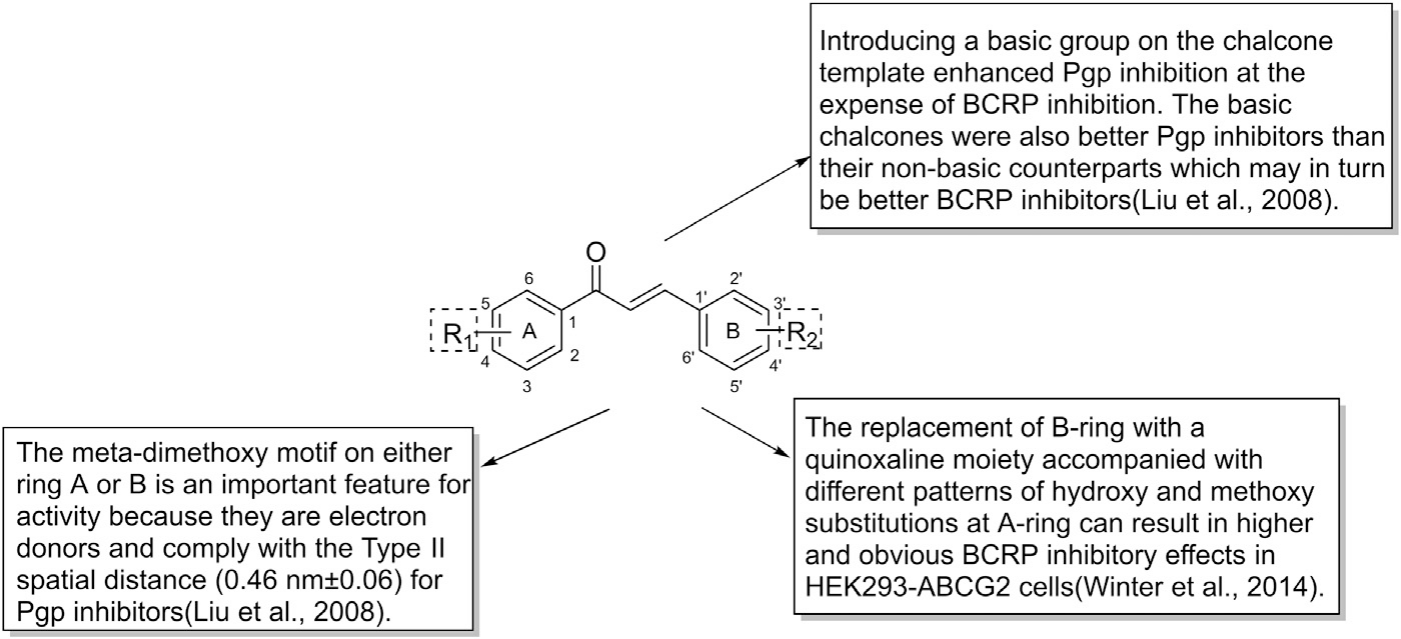
The structure-activity relationship of chalcones as ABC transporter inhibitors.

Majority of flavonoids are inhibitors of P-gp (Boumendjel et al., 2002; Falcone Ferreyra et al., 2012) and their inhibitory mechanisms are different, such as blockage of the binding site (Nabekura et al., 2008), interference with ATP hydrolysis (Shapiro and Ling, 1997), decrease of P-gp expression (Sun et al., 2013). Naringenin (1), isolated from the aerial parts of *Euphorbia pedroi*, exhibits multiple cellular functions such as antioxidant, anti-inflammatory, P-gp inhibitory activities (Chen et al., 2019). Upon applying together with felodipine in KB-V1 cells, it can decrease the P-gp expression level in KB-V1cells and increase the concentration of felodipine (Surya Sandeep et al., 2014). Quercetin (2), which is abundant in onions, apples, broccoli and berries, has a wide range of biological activities including antiproliferation and proapoptotic actions with cancer cells. Used in combination with the chemotherapeutic agent daunorubicin in gastric cancer cells, quercetin can down-regulate the ABCB1 gene, reduce the overexpression of P-glycoprotein, and inhibit the efflux of drugs. Finally, quercetin significantly sensitizes cancer cells to action of daunorubicin and increases the percentage of apoptosis (Borska et al., 2012). Another study found that methylated EGC and GC derivatives (3) exhibited better inhibitory effects targeting ABCB1 with an EC50 range from 102 to 195 nM, meanwhile they are not the substrates of ABCB1(Wong et al., 2015). Chalcones are precursors for the synthesis of flavonoids, which can also reverse multidrug resistance (Yin et al., 2019). 2′,4′-Dihydroxy-6′-methoxy-3′,5′-dimethylchalcone modulates the expression of P-gp gene. When combined with 5-fluorouracil (5-FU), it can significantly elevate tumor inhibition rate to 72.2% in BEL-7402/5-FU cell lines (Huang et al., 2012). SAR studies demonstrate that the introduction of a basic group on the chalcone moiety could enhance the P-gp inhibition and weaken the BCRP inhibition. The basic chalcones are better P-gp inhibitors, while the non-basic chalcones are better BCRP inhibitors. The good activity of chalcone is mainly related to properly placed electron donor atoms rather than lipophilicity, especially the meta-disubstituted dimethoxy motif on either ring A or B (Figure 7; Liu et al., 2008).

#### Alkaloids

Alkaloids are secondary metabolites found in plants, fungi, and bacteria. The main structural feature of alkaloids is a heterocyclic ring bearing one or more basic nitrogen. Such nitrogen atom is necessary for P-gp inhibitory activity (Qiu et al., 2014). Quinine (4), is reported to reverse doxorubin resistance in 8226/DOX6 myeloma cells and the quinine dimer can reverse the Rh123 efflux in MCF-7/DX1 cells through P-gp inhibition. Further modifications focus on the variations of the linker within the dimer and the introduction of triazole ring systems (Pires et al., 2009). Sanguinarine (5) is a benzylisoquinoline that can increase the bax/bcl2 ratio, thereby activating caspases to reverse the P-gp-induced drug resistance (Eid et al., 2012). Berberine (6) is a calcium channel blocker, which can inhibit the Wnt signaling pathway and P-gp so as to increase the intracellular accumulation of drugs (Zhang et al., 2019). Berbamine (7) can downregulate the mRNA of P-gp in imatinib-resistant BCR-ABL-positive human leukemia K562 (K562-r) cells (Wei et al., 2009). Tetrandrine (8), a bisbenzyl isoquinoline, can regulate NF-κB signaling pathway and inhibit P-gp in MCF-7/ADR cell lines when synergistically used with paclitaxel as self-assembled nanoparticles (Jiang et al., 2017). Verapamil (9), a papaverine derivative, is a classic chemosensitizer and the first found P-gp inhibitor. It can competitively inhibit the transport function of P-gp without interrupting ATP hydrolysis, and increase the intracellular accumulation of many anticancer drugs to overcome the P-gp-mediated MDR (Wang and Sun, 2020).

Indole alkaloids such as Reserpine (10), Indole-3-carbinol and indole-3-carbaldehyde can inhibit P-gp mediated efflux of drugs like doxorubicin, vincristine (Henrich et al., 2006; Wei et al., 2012). 3,4,5-trimethoxybenzyl leuconicine A (11), a derivative of leuconicine A, is a potent P-gp inhibitor as it decreases the dose of vincristine in a resistant cancer cell lines (Munagala et al., 2014).

Lamellarin O (12), isolated from southern Australian marine sponge, possesses inhibitory effects on both P-gp and ABCG2. It increases the accumulation of P-gp transporting drugs like DOX in SW620/DOX300 cells through the blockage of the binding site (Huang et al., 2014). Cyanogramide bearing a novel spirocyclic pyrrolo [1,2-c]imidazole skeleton, which is isolated from *Actinoalloteichus cyanogriseus* WH1-2216-6 can reverse the DOX-induced resistance in K562/A02 and MCF-7/DOX cells, the vincristine (VCR)-induced resistance in KB/VCR cells (Fu et al., 2014) with moderate activities in micro-molar range. A series of 5-halogenated-7-azaindolin-2-onederivatives containing a 2,4-dimethylpyrrole moiety are evaluated for their anticancer effects in MCF-7, HepG2, HT-29, A549, PANC-1, Hela. The most active one (13), IC_50_s: 4.49–15.39 μM) was proved to be even more potent than Sunitinib (IC_50_s: 4.70–>30 μM) against all tested cancer cell lines (Wang M et al., 2015). Another polysubstituted pyrrole, 4-acetyl-3- (4-fluorophenyl)- 1-(p-tolyl)-5-methylpyrrole, was found to reverse digoxin with a IC_50_ value of 11.2 µM and enhance the pharmacokinetic properties of P-gp substrates (Bharate et al., 2015). Piperine, a piperidine alkaloid from Indian spice black pepper, can downregulate the expression level of transporter ABCB1, ABCC1, and ABCG2 genes (Manayi et al., 2018). Piperine mainly inhibits P-gp activity by interacting with its nucleotide binding domain, that is to say, it competes with ATP binding site in P-gp (Singh et al., 2013). Take this into consideration, two piperine analogs Pip1 (14) and Pip2 were synthesized and exhibited better interaction with P-gp than piperine (Syed et al., 2017). Tertiary alkaloids like stemocurtisine and oxystemokerrine, isolated from *Stemona aphylla* and *Stemofoline burkillii*, also can inhibit P-gp to some extent. Stemofoline can inhibit P-gp of human cervical carcinima cell line (KB-V1) in a dose and time-dependent manner (Chanmahasathien et al., 2011).

#### Terponoids

Terponoids can be divided into several types according to the number of isoprene units within their parent structure like monoterponoids (bearing 10 C), diterponoids (bearing 20 C), sesquiterponoids (bearing 15 C), tetraterpenes (bearing 40 C). Studies have shown that lipophilic substituents at C6 position and the carbonyl group at C2, C3, C8 positions are required to make terponoids ideal P-gp inhibitors (Kumar and Jaitak, 2019).

Sooneuphanone D (15), isolated from *Euphorbia soongarica*, is a potent P-gp inhibitor with a remarkable MDR reversal activity much higher than verapamil. When sooneuphanone D is applied together with navelbine, it can significantly reduce the IC_50_ values of navelbine within KBV200 cell lines, indicating its reversal effects in P-gp overexpressed cancer cell lines (Gao and Aisa, 2017). In another study, 28 cucurbitane-type triterponoids, isolated from *Momordica balsamina* and their derivatives were studied for their collateral sensitivity effect on three different human cancer entities. Balsaminol F (16) exhibits collateral sensitivity effect through high anti-proliferative activity in gastrolic cancer cell lines (EPG85-457). Its derivatives such as balsaminagenin C exhibit reversal of multidrug resistance in human MDR1 gene-transfected mouse lymphoma cells (Ramalhete et al., 2016; Ramalhete et al., 2018). A myrsinol diterpene J196-10-1, isolated from *LANGDU*, exhibits reversal effects through competitively inhibiting P-gp transporters and increasing intracellular drug accumulation without altering MDR1 gene expression (Wang et al., 2016). Sipholenol A (17), isolated from Red Sea Sponge *Callyspongia siphonella*, mediates drug efflux activities of P-gp without altering the expression level of P-gp. Substitution of the methyl group at C15 and the oxidation of the hyroxyl group to a ketone at C4 can both cause reduced reversal activity (Jain et al., 2007; Shi et al., 2007).

## ABCG2 Inhibitors

### Natural Product

#### Flavonoids

Naringenin (1) is a common dietary flavanone which can be found in citrus fruits like oranges, bergamots and lemons (Ferreira et al., 2018). Naringenin and its derivatives were evaluated as multidrug resistance (MDR) reversers in cancer cells. The carbonyl group of naringenin was modified into hydrazones, azines, carbohydrazides which contain nitrogen atom or extra aromatic moieties. Azines and carbohydrazide derivatives exhibit potent efflux inhibition. Among them, the azine derivatives exert high inhibitory activity due to the introduction of C=N-N=C moiety and have dual inhibition on both ABCG2 and ABCC1 (Ferreira et al., 2018). Chrysin (18) shows inhibitory activities toward ABCG2 with an IC_50_ of 4.6 ± 0.5 μM, while tectochrysin and 6-prenylchrysin exhibit inhibitory activities in R482 ABCG2-transfected HEK-293 cells with an IC_50_ of 3.0 ± 0.9 μM and 0.29 ± 0.06 μM, respectively. In general, flavones inhibit ABCG2 more efficiently than flavonols, isoflavones and flavanones (Ahmed-Belkacem et al., 2005). As mentioned earlier, introducing different groups on the chalcone template can produce corresponding inhibitory effects on P-gp and BCRP. With respect to chalcone derivatives, the replacement of B-ring with a quinoxaline moiety accompanied with different patterns of hydroxy and methoxy substitutions at A-ring can result in higher and obvious BCRP inhibitory effects in HEK293-ABCG2 cells (Winter et al., 2014). The quinoxaline contributes the electrostatic interactions between the two nitrogen atoms and the ABCG2 protein (Kraege et al., 2016b). There are four key structural features that improve the ABCG2 inhibition: the *ortho*-position of the amide linker; the presence of a phenyl or 2-thienyl substitution at the amide linker; 3,4-dimethoxy substitution on ring B (Kraege et al., 2016a; Kraege et al., 2016c; Silbermann et al., 2019; Yin et al., 2019).

#### Terponoids

Guajadial (19), isolated from the leaves of Psidium guajava, is a natural meroterpenoid which has been found to have anti-tumor activity, especially in breast cancer cell lines. Guajadial has reversal effects in MCF-7/ADR and MCF-7/PTX cells by inhibiting both expressions of P-gp and ABCG2. Meanwhile, it suppresses the PI3K/Akt pathway, which is related to cell proliferation, apoptosis, and migration (Li et al., 2019).

### Tariquidar Analogs

Tariquidar (20) was an intrinsically the third-generation P-gp inhibitor which can reverse the resistance of doxorubicin, vinblastine in advanced breast cancer (Durante et al., 2017). However, due to its high toxicity in a phase III clinical trial for non-small cell lung cancer (NSCLC) (Nobili et al., 2006) and susceptibility to hydrolysis, a large number of tariquidar analogs have been synthesized to optimize its pharmacological properties. In a recent study, researchers synthesized a series of tariquidar derivatives and found that some of them are able to reverse both ABCB1 and ABCG2-mediated drug efflux, respectively. The mechanism may be related to the inhibition of ATP hydrolysis, but needs to be further verified by ATPase assay (Teodori et al., 2017). The unstable ester moiety was further replaced by ketones, which increase the stability in mouse plasma. UR-MB108 (21) and UR-MB136 (22) are the most effective ABCG2 inhibitors so far with the IC50 value about 80 nM in a Hoechst 33,342 transport assay. The molecular mechanism of their inhibitory effects lies in the depressing of ATPase by locking the inward-facing conformation (Antoni et al., 2020).

### Ko143 Analogs

The fungal toxin fumitremorgin C (23, FTC) is a specific inhibitor targeting ABCG2, however, the neurotoxicity prevents its further use (Allen et al., 2002). Later, tetracyclic analogs of FTC were developed, among which Ko143 (24) was found to be the most potent one, but it is unstable in mouse plasma and has nonspecific effects on ABCC1 and ABCB1. These have led to further structural study of ABCG2 and the development of Ko143 analogs as specific ABCG2 inhibitors (Weidner et al., 2015). Ko143 analogs are as potent as or even superior than Ko143. The modifications at C-9 position with methoxy group forms a hydrogen bond with T435 in cavity 1. The removal of methoxy groups and addition of small hydrophilic groups reduce the binding energy, while small hydrophobic groups do not make any differences. The *tert*-butyloxycarbonyl group, which can form van der Waals interactions with residues at C-3 position, also leads to decreased inhibitory effects when exchanged with ion-bearing moieties (Jackson et al., 2018).

## ABCC1 Inhibitors

### Natural Products

#### Flavonoids

Flavonoids-type compounds can also exert ABCC1 inhibitory activity in MDCKII-MRP1 cells. Chromones bearing substituted amino groups with N-substituted carboxamide moieties in C-2 are synthesized and tested for their inhibitory activities, among which (2- [4-(Benzo [c][1,2,5]oxadiazol-5-ylmethyl)piperazin-1-yl]-5,7-dimethoxy-4H-chromen-4-one (25) is proved to be the most potent ABCC1 inhibitor and stable in mouse plasma (Obreque-Balboa et al., 2016; B et al., 2020). In another study, flavonoid dimers are found to be more potent toward ABCC1 than their counterpart monomers (Dury et al., 2017). Three flavono stilbenes isolated from *Sophora alopecuroides L* were found to have an inhibitory effect toward ABCC1, which can increase the intracellular concentrations of 6-carboxyfluorescein diacetate and doxorubicin in MRP1-transfected U-2 OS cells (Ni et al., 2014). Timosaponin A-III (26, TAIII), a saponin isolated from the rhizome of *Anemarrhena asphodeloides*, were found to reverse both P-gp and ABCC1-induced drug resistance through regulation of PI3K/Akt signaling pathway (Chen J-R et al., 2016; Gergely et al., 2018). Chrysin (18), 3-methoxy-chrysin and 5,7-dihydroxy-4′-fluoro-flavone are more effective and less toxic than verapamil. Hydroxylation at different places of chrysin can alter the activity, for example, hydroxylation at C-5 or C-7 can increase GSH efflux, while hydroxylation at C-6 leads to the opposite results. However, when hydroxylation occurs both at C-5 and C-7, the activity does not increase (Lorendeau et al., 2014). In general, structure-activity relationships demonstrated that although the absence of a hydroxyl group at C-3 of flavonoid C ring is absolutely required to induce ABCC1-cell death, but it cannot to stimulate GSH efflux (Lorendeau et al., 2014).

#### Alkaloids

**FIGURE 8:**
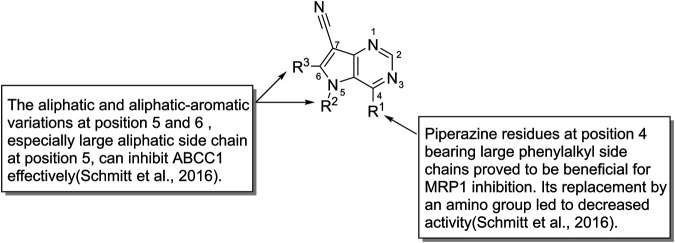
The structure-activity relationship of pyrrolo [3,2-d]pyrimidines.

The inhibitory effects of pyrrolo [3,2-d]pyrimidines toward ABCC1 show that piperazine, which bears large phenylalkyl side chains at C-4 position, can increase the inhibitory activities, whereas when piperazine is replaced with an amino group, the activity decreases. Moreover, the aliphatic and aliphatic aromatic variations in C-5 and C-6, especially the large aliphatic side chain at position 5, can inhibit ABCC1 effectively with IC_50_ value in the nanomolar range (Figure 8; Schmitt et al., 2016). A novel triazolonaphthalimide derivative named LSS-11 (9-amino-6-(2-dimethylamino)propyl]-1-(3-(dimethylamino)-propyl)benzo [de] [1,2,3]triazolo [5,4-g]isoquinoline-5,7(1H, 6H)-dione (27) acts as a potent inhibitor toward ABCC1 through DR5/PARP1 pathway and STAT3/MDR1/MRP1 STAT3 inhibition (Ji et al., 2017). Recently, a series of 9-deazapurines are synthesized (Figure 8), among which the 4-(4-(2-(1H-Indol-3-yl)ethyl)piperazin-1-yl)-5-(3-phenylpropyl)-5H-pyrrolo [3,2-d]pyrimidine-7-carbonitrile (28), has been identified to be a broad-spectrum inhibitors which regulate P-gp-mediated efflux of Calcein AM, ABCC1-mediated efflux of daunorubicin, and ABCG2-mediated efflux of pheophorbide A (Stefan et al., 2017).

### Other Inhibitors

3β-Acetyl tormentic acid (29) can reverse the resistance of doxorubicin and vincristine by mediating intracellular levels of GSH and inhibition of glutathione-s-transferase (GST) activity, instead of modulating the expression of ABCC1(Rocha Gda et al., 2014).

## Tyrosine Kinase Inhibitors

**FIGURE 9:**
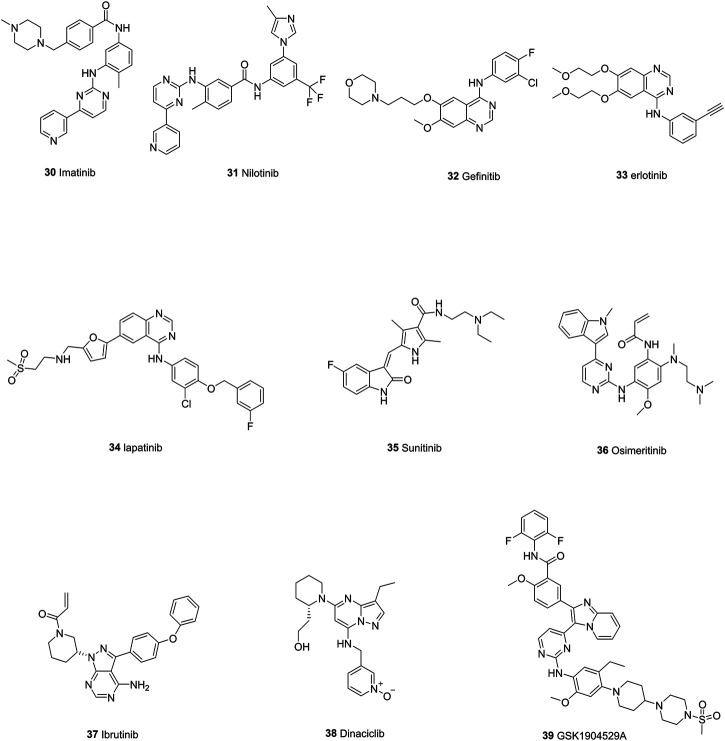
The chemical structures of representative tyrosine kinase inhibitors.

TKIs bind to the catalytic domain of tyrosine kinases and inhibit cross-phosphoralation and thereby interfere downstream signaling pathways, subsequently impairing cell proliferation and survival. The mechanism underlying the inhibitory effects of TKIs on ABC transporters may be similar to that of tyrosine kinase, that is, they compete with ATP and bind to the ATP-binding sites (Wang et al., 2015). Whether TKIs are substrates or inhibitors of ABC transporters depends on their concentrations and the cancer cells they target (Figure 9).

### Imatinib and Nilotinib

Imatinib (30) is a kinase inhibitor which targets BCR-ABL (BCR, break point cluster region; ABL, Abelson virus tyrosine kinase). Studies revealed that imatinib could reverse the drug resistance to doxorubicin by downregulating expression level of ABCB1 and subsequently resulting in accumulation of doxorubicin (Sims et al., 2013). Imatinib also exhibits inhibitory effects toward ABCG2 even at low concentration and the expression level of ABCG2 did not affect the efflux and accumulation of imatinib, which indicates that imatinib may have a higher affinity for ABCG2 than ABCB1(Ozvegy-Laczka et al., 2004). Nilotinib (31), an aminopyrimidine ATP-competitive second-generation TKI, was designed to overcome resistance to imatinib in many BCR-ABL mutants (Sacha and Saglio, 2019). It exhibits reversal effects in the doxorubicin-resistant MG63/DOX cell line (Zhou et al., 2016). Moreover, it specifically reverses mitoxantrone efflux caused by ABCG2 and increases the intracellular accumulation of mitoxantrone in over-expressing ABCG2 cells (Jordanides et al., 2006).

### Gefitinib, Erlotinib and Lapatinib

Gefinitib (32), one of the most famous EGFR inhibitors, has been used to treat NSCLC. However, enhanced ABCG2 expression has been detected within gefinitib-resistant cancer cells (Chen et al., 2011; Hegedüs et al., 2012). Inhibition of EGFR by erlotinib (33) can also induce the alteration in ABCG2 gene and protein expression level, supporting the fact that EGFR/AKT pathway is involved in the regulation of ABCG2 expression (Porcelli et al., 2014). What is more, a synergistic effect of lapatinib (34) and doxorubicin was also found in tumorspheres which generates from human breast cancer cells and exhibits drug resistance due to the overexpression of ABCB1 and increased EGFR/HER2 signaling (Lainey et al., 2012; Lainey et al., 2013).

### Sunitinib

Recently, a study has shown that in doxorubicin-resistant endothelial cell lines, the over-expression of ABCG2 and ABCB1 has a cross-resistant effect on sunitinib (35). The blockage of ABCG2 can result in a restored sunitinib cytotoxic effect (Huang et al., 2015). In another study, RCC cells with ABCG2 overexpression due to the treatment of sunitinib, were treated with elacridar, a dual inhibitor of ABCG2 and ABCB1, resulting in the restored cytotoxicity of sunitinib (Sato et al., 2015). This indicates that sunitinib is mainly transported by ABCG2 and efficient inhibition of ABCG2 is needed in sunitinib-resistant cancer cells.

### Other TKIs

There are dozens of newly found TKIs which efficiently targeted ABC transporters. For example, osimeritinib (36), a third-generation EGFR inhibitor, have been found to reverse the drug resistance within ABCB1-overexpressed bone marrow cells collected from AML patients. Evidences show that osimeritinib can increase the accumulation of Rhodamine 123 (Chen Z et al., 2016). Ibrutinib (37), an inhibitor of Bruton’s tyrosine kinase, can increase the accumulation of ABCC1 substrates within ABCC1-overexpressed HEK293/MRP1 and HL60/Adr cells (Zhang et al., 2014). Dinaciclib (38), a cyclin-dependent tyrosine kinase inhibitor, can decrease the daunorubicin efflux of MDCKII-ABCC1 and human cancer T47D cells, thus exhibit a synergistic effect when co-administrated with other anti-cancer drugs (Cihalova et al., 2015). GSK1904529A (39), an IGF-IR inhibitor, can also increase the intracellular concentration thus enhancing the cytotoxicity of ABCC1 substrate vinblastine in HEK293/MRP1 cells by inhibiting its efflux (Gupta et al., 2017). In general, TKIs have been deeply implicated in counteracting ABC-induced multidrug resistance through ways like inhibiting efflux activity, co-administration with drugs, which provides new opportunities for clinical treatment of multidrug resistance.

## Conclusion and Perspective

In recent years, many efforts have been made to modulate these ABC transporters, thus increase the intracellular concentration of drugs and reverse multidrug resistance (Kathawala et al., 2015). Several chemo-sensitizers were tested in clinical trials, like cyclosporine A, tariquidar, however, they did not show satisfying therapeutic effects due to their high toxicity, drug-drug interactions and clinical trial design problems (Robey et al., 2018). However, there are also novel strategies that can reverse multidrug resistance such as using DNA methyltransferase inhibitors (DNMTi), hypomethylating agents (HMAs) and histone deacetylase inhibitors (HDACi) (Ball et al., 2017). A DNA methyltransferase (DNMT), 5-AC (5-azacytidine) can reverse irinotecan resistance in metastatic CRC patients when combined with irinotecan (Sharma et al., 2017). In a phase II clinical trial, 17 pretreated and platinum-resistant patients with ovarian cancer were re-sensitized to carboplatin after being treated with HDACi (Matei et al., 2012). Expression of ABC transporters can also be regulated by miRNAs. For example, *ABCB1/MDR1* encoding for P-gp can be downregulated by miR-30a in advanced gastric cancer and miR-9-3p in CML to reverse drug resistance (Li et al., 2016; Li et al., 2017). miR-145 can decrease the level of *ABCC1/MRP1* and increase cisplatin toxicity in gallbladder cancer (Zhan et al., 2016). miR-490-3p regulates *ABCC2/MRP2* in ovarian cancer and possibly increases response to cisplatin (Tian et al., 2017). This provides us with an insight for finding other ways to reduce the mortality caused by multidrug resistance. More importantly, efflux of drugs has recently been found not to be the only role for ABC transporters in the failure of cancer therapy. They may also release signaling molecules, hormones, and metabolites andregulate cellular redox status, membrane lipid composition and tumor microenvironment. Additionally, MAPK, WNT, VEGF, and p53 and other signaling pathways involved in cell differentiation and proliferation should also be concerned, because they also regulate the expression and membrane localization of ABC transporters. Conclusively, the clinical failure of the ABC inhibitors makes it urgent to discover a more effective strategy.

Regarding further research on multidrug resistance, three main aspects could be focused on in future: 1) Develop a more precise drug delivery system, especially target cancer stem cells and other poorly differentiated cells. From the previous studies on cancer and multidrug resistance, we know that ABC transporters mainly over-express in poorly differentiated cells and lead to multidrug resistance. However, when drugs are delivered, they also nonspecifically target the ABC transporters of normal cells, causing many side effects. Therefore, precise delivery systems are necessary to ensure alleviation of side effects. 2) Find more about the signaling pathways related to ABC transporters. Researchers have found that signaling pathways like MAPK, WNT, VEGF, and p53 are deeply involved in regulating the expression, location of ABC transporters. Proper inhibition or activation of these signaling pathways can also reduce multidrug resistance. 3) Further investigate the molecular mechanism of ABC transporters in complex with different substrates in details by using structural biology, which provides insights in drug design and development.
